# Adult Spinal Cord Injury without Major Bone Injury: Effects of Surgical Decompression and Predictors of Neurological Outcomes in American Spinal Injury Association Impairment Scale A, B, or C

**DOI:** 10.3390/jcm10051106

**Published:** 2021-03-06

**Authors:** Bo-Ram Na, Hyoung-Yeon Seo

**Affiliations:** Department of Orthopedic Surgery, Chonnam National University Medical School and Hospital, 42 Jebongro, Donggu, Gwangju 61469, Korea; boramnaos@gmail.com

**Keywords:** spinal cord injury without radiographic abnormality, spinal cord compression, decompression, laminoplasty, magnetic resonance imaging

## Abstract

The cervical spine can be injured even in the absence of radiographic abnormality, and the best surgical treatment for adult spinal cord injury without bone injury is debated. The aim of this study was to retrospectively investigate the effect of surgical decompression for severe adult spinal cord injury without major bone injury and to establish predictors of good neurological outcome. We analyzed 11 patients who underwent surgical decompression in severe adult spinal cord injury without major bone injury patients classified as American Spinal Injury Association Impairment Scale (AIS) grade A, B, or C. Neurological assessments were performed using AIS at preoperative and postoperative 1-year follow-up. Radiological evaluations were performed using cervical magnetic resonance imaging (MRI) at preoperative. Seven cases were classified as AIS grade A; two cases as AIS grade B; and two cases as AIS grade C. Five of 11 (45.5%) patients showed improved neurological grade 1-year postoperatively. Intramedullary lesion length (IMLL) (*p* = 0.047) and compression rate (*p* = 0.045) had the most powerful effect on AIS grade conversion. This study shows that the fate of the injured spinal cord is determined at the time of the injury, but adequate decompression may have limited contribution to the recovery of neurological function. Compression rate and IMLL on MRI can be used as a predictor of neurological recovery.

## 1. Introduction

Cervical spinal cord injuries represent 20–33% of all spinal cord injuries, and most often, spinal cord injuries occur at the subaxial level [1]. In recent years, cervical spinal cord injury has been increasing in the elderly population with degenerative changes, even without bony injury such as spinal fracture or dislocation. The terminology regarding this type of injury remains controversial, but we adopted the term spinal cord injury without major bone injury [2,3].

Generally, for spinal cord injury resulting from fracture or dislocation of the spinal column, surgery consisting of decompression and stabilization is the treatment of choice [4]. Controversy exists with regard to the choice of surgery or conservative treatment for adult spinal cord injury without major bone injury, because it usually does not require spinal column reconstruction surgery. Nonsurgical treatments such as steroid therapy, fixation, and avoiding activities are the primary treatment options for patients with adult spinal cord injury without major bone injury. However, when conservative treatment alone is considered insufficient, surgical decompression should be considered as another option for patients with clinical and MRI evidence of severe spinal cord injury. The efficacy of surgical decompression to reduce secondary cord injury is controversial [5], but adequate decompression may have neurological recovery potential.

Although adequate decompression is achieved through decompression surgery, neurological recovery appears to varying degrees. Efforts have recently begun to establish the predictors of neurological outcome. We aimed to investigate the effect of surgical decompression with expansive laminoplasty for severe adult spinal cord injury without major bone injury and to establish predictors of good neurological outcome.

## 2. Materials and Methods

### 2.1. Study Population

This study retrospectively analyzed 11 patients who underwent surgical decompression with expansive laminoplasty for adult spinal cord injury without major bone injury from 2003 to 2019. This study was performed after approval from the institutional review board (CNUH-2020-288). Subjects were eligible based on the inclusion criteria: age > 18 years; patients classified as American Spinal Injury Association Impairment Scale (AIS) grade A, B, or C; without bone injury (spinal fracture or dislocation) on plain radiography and computed tomography (CT) of the cervical spine; and spinal cord edema or hemorrhage seen on cervical magnetic resonance imaging (MRI). Subjects were excluded based on the following conditions: patients classified as AIS grade D or E; bone injury (spinal fracture or dislocation) observed on plain radiography and CT of the cervical spine; and almost normal images on cervical MRI. Patients with degenerative changes were not excluded. All patients arriving within 8 h of trauma received high-dose methylprednisolone. Preoperative and 1-year postoperative follow-up neurological assessments were carried out on all patients and were graded according to the AIS, and their motor function and sensory points evaluated according to the AIS score [6]. The AIS motor score (AMS) was mainly based on the motor function score of the 10 pairs of key muscles in the upper and lower limbs, with 5 points for each muscle and 100 points in total. The AIS sensory score (ASS) was mainly based on the pin-prick and light touch scores in each of the 28 key sensory dermatomes, with 2 points for each dermatome and 112 points in total.

### 2.2. Preoperative Physical Examination and Imaging Examination

Immediately after arriving at the hospital, all patients were evaluated by the emergency specialist, the trauma team, and the spine surgeon. If no other neurological or medical condition precluded the neurological evaluation, the first AIS assessment was done at that time point. Complete radiological evaluation, including plain radiography, CT, and MRI, was performed to assess compression and injury in the spinal cord.

MRI scans were analyzed following a standardized protocol. Intramedullary findings were classified as either edema or hemorrhage in sagittal T1- and T2-weighted MRI images: edema was defined as hyperintense T2 signals (central and peripheral) and normal T1 signal, and hemorrhage was defined as inhomogeneous T1 and hypointense (central) T2 signals with a hyperintense rim [7].

**Figure 1:**
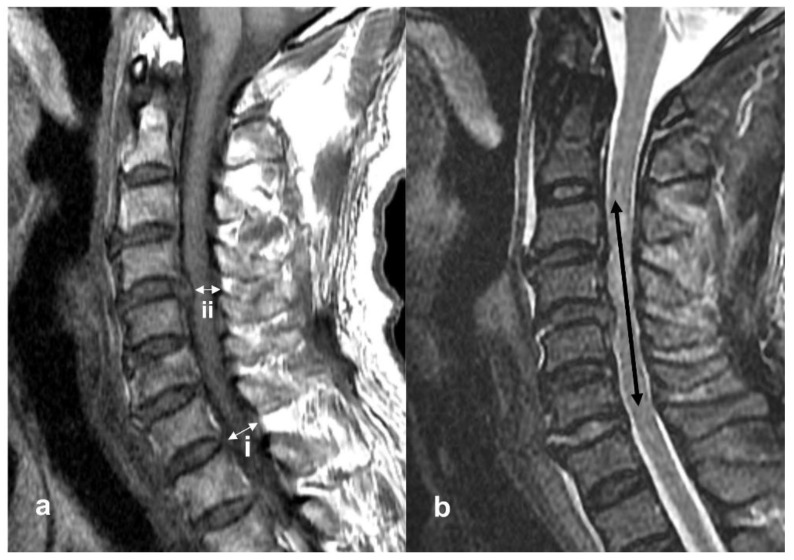
Sagittal magnetic resonance imagery (MRI) obtained in a 69-year-old man who presented with severe adult spinal cord injury without major bone injury and American Spinal Injury Association Impairment Scale (AIS) grade A. (**a**) T1-weighted image. (i) The diameter of the cervical cord at the non-compression level. (ii) The diameter of the cervical cord at the injured level. Compression rate was calculated by the equation. Compression rate was 35.2%. (**b**) T2-weighted image at C3-4-5-6 showed the signal change, and the rostrocaudal length of damage was measured in millimeters. Intramedullary lesion length (IMLL) was 62 mm.

The rate of spinal cord compression was measured using sagittal view MRI (Figure 1a). The spinal cord diameter was measured at both the non-compressed level and the injured level on T1-weighted MRI images, and the compression rate was calculated using the following equation:

(i − ii)/I × 100%

where (i) is the diameter of the cervical cord at the non-compression level and (ii) is the diameter of the cervical cord at the injured level [8].

Intramedullary lesion length (IMLL) was defined as the rostrocaudal length of high-signal intensity from the injury epicenter, measured in millimeters (Figure 1b). IMLL was measured on T2-weighted images [9].

### 2.3. Steroid Protocol

Methylprednisolone was administered according to the recommended National Acute Spinal Cord Injury Study (NASCIS) II and III protocols. Infusion was started within 8 h of injury. An initial IV bolus of 30 mg/kg over 15 min was infused, and after a pause of 45 min, 5.4 mg/kg/h for 23 h (if started within 3 h after trauma) or for 48 h (if started at 3 to 8 h after trauma) was infused. All patients included in this study received methylprednisolone.

### 2.4. Surgical Procedure

**Figure 2:**
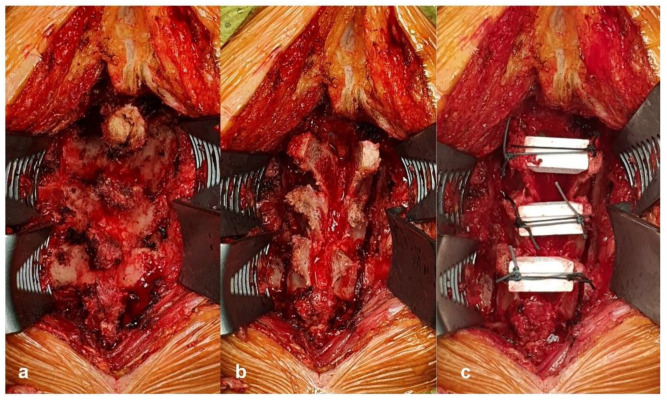
Surgical procedure technique in this study. (**a**) Cervical laminae are exposed laterally to the medial aspect of the facet joints. (**b**) The spinous processes are split sagittally, and bilateral gutters for the hinges are made. (**c**) The split laminae is opened bilaterally, and a hydroxyapatite spacer is placed inside.

We adopted the surgical procedure of surgical decompression with expansive laminoplasty using the double-door technique. A posterior approach was made along the nuchal ligament to the line of the spinous processes. Cervical laminae were exposed laterally to the medial aspect of the facet joints, and the interspinous ligaments were removed. The spinous processes were split sagittally with an air-drill or a surgical ultrasonic knife. After bilateral gutters for the hinges were carefully made with a high-speed burr at the transitional area between the facet joint and laminae, spinal canal enlargement was achieved by opening the split laminae bilaterally with a spreader and placing a hydroxyapatite spacer or allograft held in place by two non-absorbable threads (Figure 2).

### 2.5. Statistical Analysis

All statistical analyses were performed using the SPSS software ver. 22.0 (IBM Corp, Armonk, NY, USA). We report descriptive statistics as the mean and standard deviation for continuous variables and as proportions for categorical variables. We conducted a univariate analysis to assess the association between each independent variable and AIS grade conversion using the Mann–Whitney U test or Fisher’s exact test. The Wilcoxon signed-rank test was used for preoperative and 1-year follow-up comparisons. *p* value < 0.05 was considered statistically significant.

## 3. Results

A total of 11 (all male) adult patients with spinal cord injury without major bone injury with a mean age of 63.8 years (range, 50–81 years) were included. Among them, four cases were caused by car accidents and seven cases were caused by fall. In this study, seven cases were classified as AIS grade A; two cases as AIS grade B; and two cases as AIS grade C. All patients underwent cervical plain radiography, CT, and MRI imaging before surgery. Radiological evaluation revealed cervical spondylosis (CS) in six patients and ossification of the posterior longitudinal ligament (OPLL) in two patients. All 11 patients hospitalized within 48 h were operated on within 72 h. The cervical MRI showed spinal cord edema in six cases and spinal cord hemorrhage in five cases. The minimum follow-up period was 1 year (range, 12–23 months). The detailed demographic and clinical characteristics of the study are shown in Table 1.

Five patients out of 11 (45.5%) showed improved neurological function 1-year postoperatively (Table 2). Neurological improvement of one or more AIS grades was observed in three patients (42.9%) of AIS grade A, of whom one converted to AIS grade B and 2 to AIS grade C. Similarly, 1 patient (50.0%) of AIS grade B converted to AIS grade C, and 1 patient (50.0%) of AIS grade C converted to AIS grade D, while none showed neurological deterioration. The effect of eight independent variables on AIS grade conversion was analyzed (Table 3). Among the demographic variables, age (*p* = 0.464), time from injury to surgery (*p* = 1.000), and MRI type (*p* = 0.242) did not affect AIS grade conversion. In addition, neurological status at admission, including admission AIS grade (*p* = 1.000), and admission AMS (*p* = 0.359) and admission ASS (*p* = 0.854), did not affect AIS grade conversion. IMLL on T2 images (*p* = 0.047) and spinal cord compression rate on T1 images (*p* = 0.045) had the most powerful effect on AIS grade conversion.

Changes in the AMS and ASS were investigated from admission to 1-year postoperatively. At the 1-year follow-up, AMS (*p* = 0.005) and ASS (*p* = 0.007) were significantly higher than those before surgery (Table 4). We divided the 11 patients into preoperative and 1-year follow-up groups according to their MRI type, compression rate, and IMLL. According to the MRI type, only AMS was significantly improved in the edema type (*p* = 0.028). According to the compression rate, both AMS (*p* = 0.035) and ASS (*p* = 0.035) significantly improved at compression rates below 20%. According to the IMLL, AMS (*p* = 0.035) and ASS (*p* = 0.035) were significantly improved when the IMLL was below 50 mm (Table 5).

## 4. Discussion

The results of this study show that adequate decompression may have limited contribution to the recovery of neurological function. Despite several reported basic and clinical studies, effective treatment for cervical spinal cord injury has not been established. Whether surgical decompression for severe cervical spinal cord injury has better outcomes than conservative treatment remains controversial. Some surgeons have reported improvement in paralysis after spinal cord decompression [10,11,12], whereas others have concluded that the fate of an injured spinal cord cannot change by decompression surgery [8,13]. In this study, neurological improvement of one or more AIS grades was observed in 45.5% of patients at 1-year postoperatively. Compared to results reported in other studies, decompression surgery is better at AIS grade conversion rates than conservative treatment (20%) [14] or inadequate decompression (18.5%) [15]. This study showed good outcomes (45.5%) after surgical treatment, but has limited implications because it has not been compared to conservative treatment.

In adult spinal cord injury without major bone injury, patients whose spinal cords were compressed by degenerative changes require decompression surgery to help recover from paralysis. Despite the controversy about the efficacy of surgical treatment, most doctors agree that adequate decompression is the primary criterion for choosing surgical procedures regardless of the type of surgery. The choice of bony decompression surgical method varies. Currently, cervical laminoplasty are broadly divided into two types based on the site of osteotomy: the open-door type (at one side of the lamina-facet junction) [16] and the double-door type (at the central spinous process and lamina) [17]. While each method has its own advantages and disadvantages, both are effective in expanding the compressed spinal canal. We adopted expansive laminoplasty using the double-door technique for adequate decompression in severe adult spinal cord injury without major bone injury.

Even if adequate decompression is achieved through decompression surgery, neurological results can be found to varying degrees. Recent efforts have begun to establish the predictors of neurological outcome, including injury type (edema or hemorrhage), compression rate, and IMLL on MRI. Kulkarni et al. [7] described three imaging patterns of intramedullary abnormalities (cord hemorrhage, edema, and mixed type), and their prognostic value has been the focus of a number of reports [18,19]. Kawano et al. [8] defined a cutoff point for the spinal cord compression rate. A 20% compression rate is a point at which many researchers judged the spinal cord to be compressed, and therefore, considered as needing decompression surgery. Aarabi et al. [15,20] noted potential predictors of AIS grade conversion after surgical decompression, and these studies showed that IMLL was the strongest variable that correlated with AIS grade conversion. In our study, IMLL and compression rate had the most powerful influence on improved AIS grade conversion at 1-year after surgery. If IMLL already appeared at a long level on MRI and the compression rate was high, decompression by laminoplasty did not achieve good neurological results. On the other hand, when IMLL appeared at a short level on MRI and the compression rate was low, decompression by laminoplasty achieved better neurological results. Based on these results, it seems that the severity at the injury stage determines the fate of the injured spinal cord and that decompression surgery has only a limited role in the fate of the injured spinal cord.

This study has some limitations. First, this study evaluated a relatively small number of cases and had short-term follow-up. Because the incidence of adult spinal cord injury without major bone injury is rare, only a few cases were included. However, since our study was a single-center study, this may reduce the impact of variability in management associated with different centers. Second, this study reported the results of surgical decompression, but the results could not be compared with conservative treatment. The findings reported here will require verification in a comparative, prospective study.

## 5. Conclusions

This study shows that the fate of the injured spinal cord is determined at the time of the injury, but adequate decompression may have limited the contribution to the recovery of neurological function. Neurological recovery was only promoted limitedly in severe neurological grade patients with mild cervical lesions on MRI. Cervical MRI can be used as a predictor of neurological recovery; especially, compression rate and IMLL on MRI were significantly correlated with AIS grade conversion. Therefore, we recommend careful consideration of surgical decompression in patients with spinal cord injury without major bone injury, with low compression rates and short levels of IMLL on MRI.

## Figures and Tables

**Table 1:** Demographic and clinical data of adult spinal cord injury without major bone injury patients.

Cases	Sex/Age	Injury Mechanism	Degeneration (CS or OPLL)	Time from Injury to Surgery (h)	MRI Type	Compression Rate	Intramedullary Lesion Length	Operation Level	AIS Grade
1	65/male	Car accident	CS	<72	hemorrhage	34.6%	50 mm	C4-7	A->A
2	68/male	Fall down	OPLL	<24	edema	16.8%	42 mm	C3-5	B->C
3	50/male	Car accident	-	<48	hemorrhage	10.5%	59 mm	C4-7	B->B
4	79/male	Fall down	CS	<24	edema	18.5%	55 mm	C3-6	C->D
5	69/male	Fall down	OPLL	<24	hemorrhage	35.2%	62 mm	C4-6	A->A
6	81/male	Fall down	CS	<12	edema	27.8%	41 mm	C4-6	C->C
7	50/male	Fall down	CS	<24	hemorrhage	23.9%	71 mm	C4-6	A->A
8	55/male	Car accident	CS	<12	edema	32.9%	66 mm	C3-6	A->A
9	57/male	Car accident	CS	<12	hemorrhage	19.8%	42 mm	C3-5	A->B
10	62/male	Fall down	-	<12	edema	9.4%	20 mm	C3-4	A->C
11	66/male	Fall down	CS	<72	edema	13.2%	44 mm	C4-5	A->C

CS: cervical spondylosis; OPLL: ossification of posterior longitudinal ligament; AIS: American Spinal Injury Association Impairment Scale; MRI, magnetic resonance imaging.

**Table 2:** Preoperative and 1-year follow-up AIS grade.

Preoperative AIS Grade	1-Year Follow-up AIS Grade				
	A	B	C	D	E
A (*n* = 7)	4	1	2	0	0
B (*n* = 2)	0	1	1	0	0
C (*n* = 2)	0	0	1	1	0

AIS: American Spinal Injury Association Impairment Scale.

**Table 3:** AIS grade conversion in various categories of the study.

Category	AIS Not Converted (*n* = 6)	AIS Converted (*n* = 5)	*p* Value
Age	61.7 ± 12.3	66.4 ± 8.2	0.464
Admission AIS grade			1.000
A	4	3	
B	1	1	
C	1	1	
Admission AMS	21.0 ± 9.4	13.4 ± 14.9	0.359
Admission ASS	56.8 ± 22.9	85.6 ± 86.2	0.854
Time from injury to surgery			1.000
<12 h	2	2	
12–24 h	2	2	
>24 h	2	1	
MRI type			0.242
Edema	2	4	
Hemorrhage	4	1	
Compression rate (%)	27.4 ± 9.4	15.5 ± 4.0	0.045 *
IMLL (mm)	58.2 ± 11.0	40.6 ± 12.7	0.047 *

AIS: American Spinal Injury Association Impairment Scale; AMS: American Spinal Injury Association Impairment Scale Motor Score; ASS: American Spinal Injury Association. Impairment Scale Sensory Score; IMLL: Intramedullary lesion length; * *p* value < 0.05.

**Table 4:** Preoperative and 1-year follow-up AIS score.

AIS Score	Preoperative	1-Year Follow-up	*p* Value
AMS	17.5 ± 12.2	30.6 ± 19.3	0.005 *
ASS	69.9 ± 58.8	117.0 ± 62.7	0.007 *

AIS: American Spinal Injury Association Impairment Scale; AMS: American Spinal Injury. Association Impairment Scale Motor Score; ASS: American Spinal Injury Association. Impairment Scale Sensory Score; * *p* value < 0.05.

**Table 5:** Preoperative and 1-year follow-up AIS scores according to MRI type, compression rate, and IMLL.

	Preoperative	1-Year Follow-up	*p* Value
Edema			
AMS	18.7 ± 12.4	38.8 ± 17.8	0.028 *
ASS	69.9 ± 58.8	117.0 ± 62.7	0.052
Hemorrhage			
AMS	16.2 ± 13.2	20.8 ± 17.7	0.090
ASS	40.0 ± 15.0	73.6 ± 31.1	0.057
Compression rate ≤ 20%			
AMS	14.8 ± 13.8	35.8 ± 22.8	0.035 *
ASS	78.0 ± 79.3	131.0 ± 58.6	0.035 *
Compression rate > 20%			
AMS	20.8 ± 10.5	24.4 ± 13.8	0.057
ASS	60.2 ± 23.8	100.2 ± 69.9	0.090
IMLL ≤ 50 mm			
AMS	14.8 ± 13.2	34.3 ± 22.8	0.035 *
ASS	59.3 ± 37.2	134.3 ± 53.0	0.035 *
IMLL > 50 mm			
AMS	20.8 ± 11.3	26.2 ± 15.2	0.057
ASS	82.6 ± 81.0	96.2 ± 73.0	0.090

AIS: American Spinal Injury Association Impairment Scale; AMS: American Spinal Injury. Association Impairment Scale Motor Score; ASS: American Spinal Injury Association. Impairment Scale Sensory Score; IMLL: Intramedullary lesion length; * *p* value < 0.05.
